# Impedimetric Single Carbon Fiber Electrode for Ultrasensitive Detection of *Staphylococcus aureus* Pathogen DNAs in Breast Milk by CRISPR Technology

**DOI:** 10.1021/acsomega.4c02738

**Published:** 2024-05-24

**Authors:** Hilmiye Deniz Ertuğrul Uygun, Dilek Odaci

**Affiliations:** † Center for Fabrication and Application of Electronic Materials, Dokuz Eylül University, Buca, İzmir 35220, Türkiye; ‡ Faculty of Science, Department of Biochemistry, Ege University, Bornova, İzmir 35040, Türkiye

## Abstract

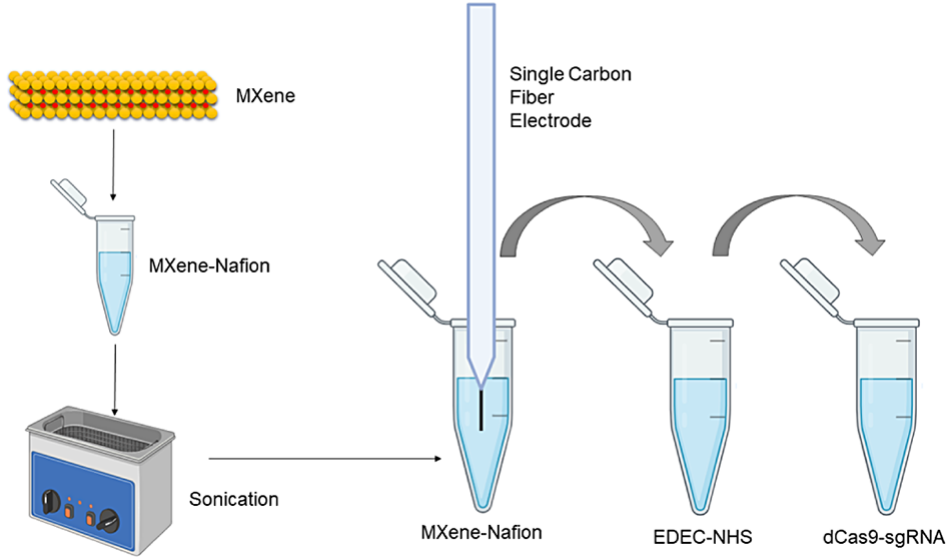

This study introduces a novel biosensing approach for the detection of pathogen DNA in breast milk utilizing single carbon fiber electrodes (SCFE) enhanced with MXene nanomaterial layers. The primary innovation lies in the modification of SCFE with MXenes to increase the electrode’s surface area, followed by surface activation for the immobilization of dCas9-sgRNA complexes. This modification aims to leverage the unique properties of MXenes and the selective binding capability of the CRISPR technology for efficient and specific pathogen detection. Electrochemical impedance spectroscopy (EIS) and scanning electron microscopy (SEM) analyses were employed to characterize the electrode modifications and the immobilization process, demonstrating the successful enhancement of biosensor performance. This study further optimized the chronoimpedimetric detection method to achieve rapid, sensitive, and selective detection of *Staphylococcus aureus* (SAu) DNA in breast milk, with a notable detection time of 60 s in real samples. The biosensor demonstrated high selectivity and sensitivity, with a linear detection range between 50 and 6000 fM and a limit of detection (LOD) of 14.5 fM. The reproducibility and stability of the biosensor were also confirmed through multiple tests, showing promising potential for clinical and public health applications.

## Introduction

Breast milk is widely recognized as the optimal source of nutrition for infants, offering a range of benefits for health, growth, immunity, and development. It contains the perfect balance of nutrients, vitamins, and minerals essential for a newborn’s growth and development.^1^ Additionally, breast milk is packed with antibodies and other immune factors that help protect infants against infections and diseases during their early life, when their immune system is still developing.^2−4^ However, the presence of pathogens in breast milk can pose significant health risks to infants. Pathogens are disease-causing microorganisms such as bacteria, viruses, and parasites.^5,6^ When present in breast milk, these pathogens can be transmitted to the infant, leading to various infections and illnesses.^7−9^ This is particularly concerning for newborns and young infants, whose immune systems are not fully developed and are more susceptible to infections. Infections in breast milk and the presence of pathogens are critical concerns in neonatal care and maternal health. Breast milk, while being the most nutritious and beneficial source of nourishment for infants, can sometimes become a medium for the transmission of infectious agents. Understanding the types of pathogens that can contaminate breast milk and the implications of such contamination is essential for ensuring the health and safety of both infants and breastfeeding mothers. Infants who consume contaminated breast milk can suffer from various infections, ranging from mild gastrointestinal issues to more severe systemic infections.^10^ Newborns, especially preterm or immunocompromised infants, are at a higher risk due to their underdeveloped immune systems. The detection of pathogens in breast milk can sometimes lead to mothers being advised to stop breastfeeding, which can disrupt the natural feeding process and bonding experience. In cases where pathogen transmission through breast milk is a concern, medical intervention may be necessary. This could include antiviral therapies, antibiotics, or alternative feeding strategies for the infant. Proper breast hygiene, sterilization of feeding equipment, and safe milk storage practices are crucial in preventing contamination. For mothers with known infections, medical guidance is essential to assessing the risks and benefits of breastfeeding. Regular screening for infections in breastfeeding mothers, especially in high-risk populations, is important for the early detection and prevention of transmission. The topic of pathogens in breast milk, particularly focusing on bacteria such as *Staphylococcus aureus* and *Streptococcus agalactiae*, is of significant importance in the context of infant health and breastfeeding safety.^11^ Both of these bacteria are common causes of infection and can be transmitted through breast milk, posing risks to both the nursing infant and the mother. *Staphylococcus aureus*, commonly found on the skin and in the nasal passages, can contaminate breast milk if there are cracks in the nipple or if breast pumps and storage containers are not properly sterilized^12−15^ Health Implications for Infants exposed to *Staphylococcus aureus* through breast milk can develop a range of infections, from minor skin conditions such as pimples and impetigo to more severe issues such as pneumonia, meningitis, or sepsis, especially in premature or immunocompromised babies. Mothers can develop mastitis, a painful inflammation of breast tissue that is often caused by a *Staphylococcus aureus* infection. Symptoms include breast pain, swelling, warmth, fever, and chills. Management and Treatment: Treatment typically involves antibiotics to clear the infection. In cases of mastitis, it is often recommended to continue breastfeeding or pumping to clear the milk ducts, alongside medical treatment. *Streptococcus agalactiae*, or Group B *Streptococcus* (GBS), is commonly found in the gastrointestinal and genitourinary tracts.^16,17^ It can be transmitted to the infant through breast milk, although this is less common than transmission during childbirth. For infants, GBS can lead to serious infections, such as meningitis, sepsis, and pneumonia. Newborns are particularly vulnerable due to their immature immune systems. Pregnant women are typically screened for GBS colonization to manage the risk during delivery. However, less emphasis has been placed on the risk of transmission through breast milk. If an infant is diagnosed with a GBS infection, they are usually treated with antibiotics. In some cases, mothers with GBS may also receive antibiotics. The ability to detect pathogens in breast milk at an early stage is vital. It ensures that contaminated milk is not consumed by infants, thereby preventing potential illnesses. For mothers who donate breast milk to milk banks, it is essential to ensure that the milk is free from pathogens to safeguard the health of the recipient infants. Typically, the presence of bacteria in milk is identified by culturing the bacteria on agar plates and counting the resulting colonies. This traditional method is time-consuming, requiring overnight incubation and precise colony counting. Alternatively, quicker detection methods such as PCR (polymerase chain reaction), ELISA (enzyme-linked immunosorbent assay), and ELFA (enzyme-linked fluorescent assay) are available. Immunoassays offer advantages in speed, cost, and simplicity, making them suitable for initial screenings before conducting more detailed PCR analyses.^18^ The specificity of PCR lies in its ability to target genes associated with specific bacteria. Advanced PCR techniques can detect as little as 5 CFU/mL within 20 min. Additionally, the DNA-based nanobarcode method provides rapid results.^19^ Understanding the types and concentrations of pathogens that can contaminate breast milk is important for developing better preservation and sterilization techniques. The development of sensitive and rapid biosensors for pathogen detection in breast milk is critical for ensuring the health and safety of infants. This necessity arises from the potential risk of transmitting infections through breast milk, which can lead to severe health complications in infants. Early detection of pathogens in breast milk not only prevents the consumption of contaminated milk but also aids in the improvement of milk preservation and sterilization techniques, crucial for milk banks that provide donated milk to infants in need. In this context, our study introduces a novel biosensor based on single carbon fiber electrodes (SCFE) modified with MXene layers for the enhanced detection of pathogen DNA in breast milk. By leveraging the increased surface area provided by MXene modifications and the selective binding capabilities of dCas9-sgRNA, our biosensor demonstrates remarkable sensitivity and specificity in detecting SAu DNA. Electrochemical impedance spectroscopy (EIS) and scanning electron microscopy (SEM) analyses confirm the successful modification of the electrode surface and immobilization of biological molecules, leading to rapid detection times and high selectivity.^20^ The discovery of MXenes, defined as a family of 2-dimensional layered compounds, has attracted great attention in the scientific community due to their structural and electronic properties.^21^ MAX phases are the primary precursor materials used in the synthesis of MXenes. In their generalized formula, Mn + 1AXn, A can be any element from IIIA or IVA groups, and X can be either carbon or nitrogen; however, combinations that result in the production of carbonitride are also feasible, and *n* = 1 – 4. Graphene is less appealing than MXene because it lacks control over the material’s properties. Whereas graphene permits functionalization only, this can be accomplished through processing, doping, and functionalization. It was discovered that adding different functional groups, altering the spacing between layers, doping in the location of M, A, or X in the corresponding MAX phase, and modifying their composition can all be used to influence the properties of MXene.^22^

The study not only presents a promising approach for pathogen detection in breast milk but also sets the stage for future research aimed at broadening the range of detectable pathogens, improving biosensor integration into portable devices, and enhancing the overall utility of biosensors in clinical and environmental applications.

In conclusion, we used a single carbon fiber electrode as a working electrode to obtain more sensitive signals and modified it by MXenes by using Nafion to immobilize on the surface to increase surface area; dCas9-sgRNA complex was immobilized on it covalently to construct our biosensor.

## Experimental Section

### Reagents

All consumables were obtained from Merck (USA). dCas9 proteins were obtained from Applied Biological Materials Inc. (ABM) (Canada). sgRNAs were obtained from Synthego (USA). Carbon microelectrodes (DRP-110) were constructed using carbon fiber cables from Metrohm Dropsens AG (Switzerland). Working and auxiliary electrodes were made of carbon, while the reference electrode was silver/silver chloride. *Staphylococcus aureus* (SAu) and *Streptococcus agalactiae* (SAg) pathogen DNAs were also obtained as detection DNAs.

### Instrumentation

All electrochemical detections were carried out by PalmSens EmStat4S Potentiostate (Netherlands) with a PSTrace 5.9 interface. A scanning electron microscope (JEOL JSM-7400F) was used to examine the morphology of the microelectrodes. Raman analysis was performed with a Renishaw inVia Raman spectrometer with 514 nm laser excitation. Thermo Scientific NICOLET iS10 instrument was used for FT-IR analysis. The elemental composition and surface chemistry of the MXenes were determined by X-ray photoelectron spectroscopy (XPS, Thermo Scientific K-Alpha) with a beam size of 400 μm diameter by using a monochromatic 1486.7 eV Al–Kα X-ray source.

### Carbon Fiber Electrode, MXene Production, and Carbon Fiber Electrode Modification

The carbon fibers inside the industrially used carbon fiber cables were cut into certain lengths (at least 10 cm) and separated one by one with micro forceps under the microscope. Then, the separated microfibers were placed in a 10 cm glass capillary, and the tube and carbon fiber structure were fused in the capillary extender (a device that thins and lengthens the capillary tube by applying heat and pulling it). Then, 10 mm glass was broken from the tip under the microscope with micro forceps, and the fiber part was exposed. Epoxy was applied to the broken part, and the carbon fiber was pulled into the vacuum from the other end. With this process, the part of the electrode that will enter the liquid part is insulated, and the carbon fiber is fixed. On the other end, one end of a copper cable was treated with Ag/AgCl paste and placed in the capillary to ensure contact with the fiber. When in contact with the fiber, the carbon electrode production was completed by rotating it around itself, maximizing the copper–carbon contact, and gluing the exposed copper tip with epoxy, and the electrode was left to dry at room temperature for at least 3 days.

MXene production was performed by following the protocols.^23^ Hydrogen fluoride (HF) as the etching agent was used for MXene production. Titanium aluminum carbide (Ti_3_AlC_2_ (1 g)) was dispersed in 40%, 30 mL HF inside of a plastic wide round-bottom flask with a closed cap and vigorously stirred for 24 h at room temperature to form the MAX structural phase. That phase was then taken into the falcon tube and purged nitrogen for 5 min to remove oxygen to prevent oxidation centrifuged 5 times at 3500 rpm and washed with pure water to remove acid, increase pH, and avoid impurities. The pellet was then dissolved in dimethyl sulfoxide (DMSO) mixed for 24 h for delamination and centrifuged again; the pellet was dried under a vacuum. The dried powder was then investigated by scanning electron microscopy to investigate layers.

The modification of the electrode was carried out using a noncovalent modification of the electrode. This was the hardest part of the study; therefore, gentle and slow modification is encouraged to avoid electrode loss. First of all, MXene (1 mg/mL) was dissolved in an Eppendorf tube with Nafion and stirred by using a vortex. 100 μL of this solution was added to a 200 μL Eppendorf tube, and then the electrode soaked into this Eppendorf tube stabilized with a clamp. This incubation was carried out for 2 h. To avoid precipitation of the MXenes, every 15 min, the Eppendorf tube vortexed and placed again. The modified electrode was then placed in a 40 mM/10 mM EDC/NHS (*N*-ethyl-*N*′-(3-(dimethylamino)propyl)carbodiimide/*N*-hydroxysuccinimide) solution to activate carbonyl groups to form covalent immobilization for 30 min.^24^ The activated electrode was then soaked in 1 mg/mL dCas9-sgRNA complex to immobilize the biorecognition receptor for 15 min. The performances of the electrode and the immobilization layers were tested by using electrochemical impedance spectroscopy (EIS). The EIS parameters were optimized as follows 50 000–0.1 Hz frequency by applying 200 mV DC and 5 mV AC potential. All electrochemical experiments were carried out under room temperature conditions. EIS measurements were performed by using a 5 mM Fe(CN)_6_^3-/4–^ redox probe including 100 mM KCl (potassium chloride) at pH = 7 50 mM phosphate buffer. dCas9 and pathogen DNA binding procedure was performed between 20 and 38 °C. Pathogens were added to the breast milk samples as standards. Real samples were used for the matrix effect, real sample analysis, repeatability, and selectivity studies. After the biosensor modifications, the biosensor was tested by using chronoimpedimetric (CI) detection to obtain the SAu DNA detection time. CI detection was carried out in breast milk, and SAg and Sau DNAs were added. Chronoimpedance parameters were set as 150 mV AC, 5 mV AC, and 600 Hz for 400 s. Afterward, six biosensors were prepared for storage stability, and three electrodes were kept in a humidified and dark place (to prevent biological growth); the others were kept at +4 °C. 50 fM real samples were measured with these biosensors, and the signal decreased compared with *t* = 0.

## Results and Discussion

**Figure 1:**
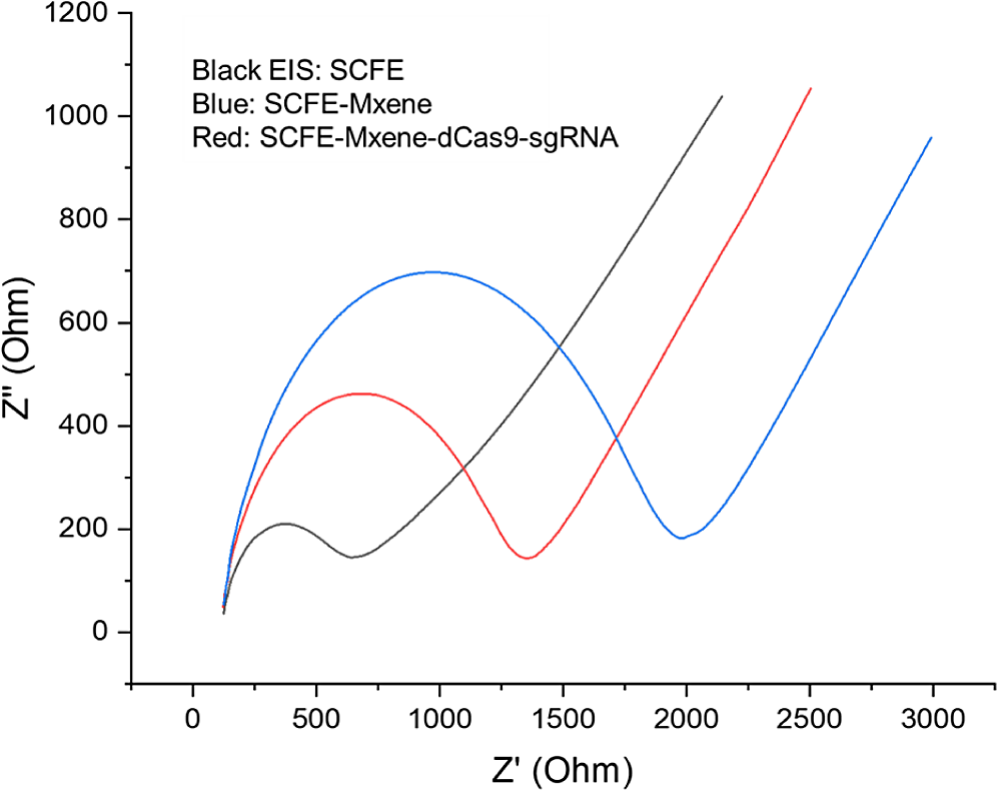
Single carbon fiber electrode modification layer investigation by EIS (black EIS: SCFE, blue: SCFE-MXene, red: SCFE-MXene-dCas9-sgRNA).

**Figure 2:**
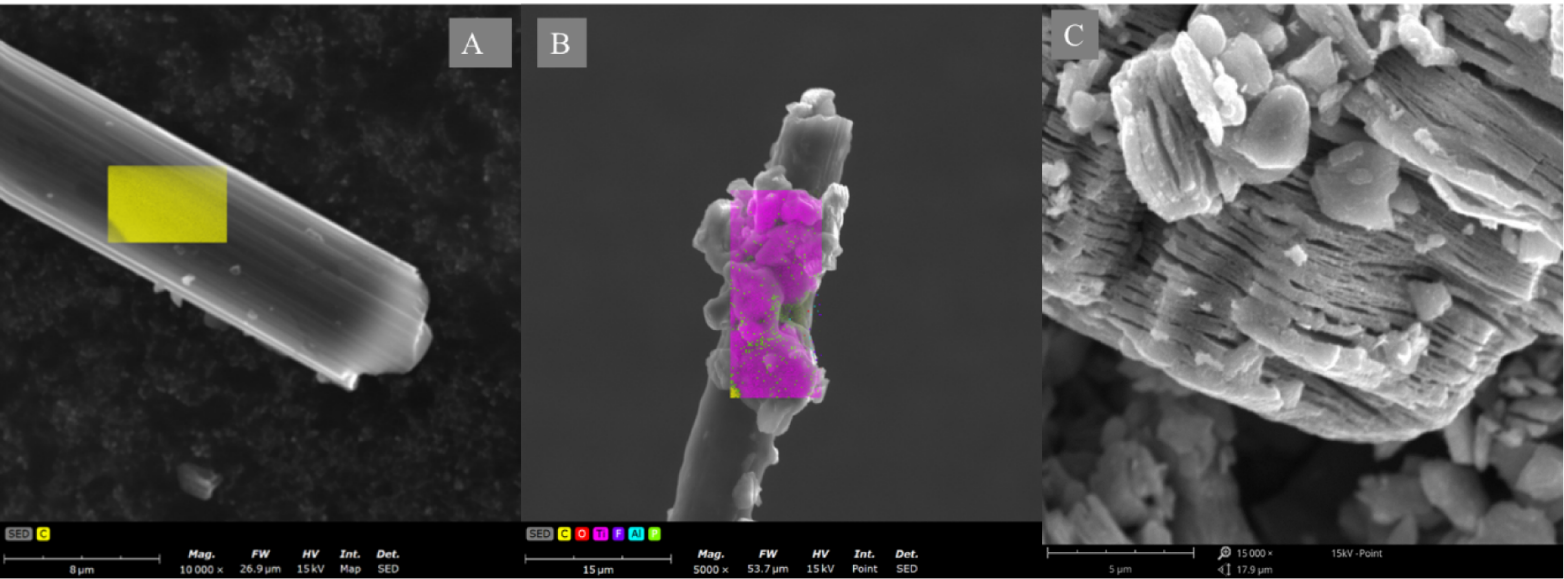
SEM and EDX analysis of the modified single carbon fiber electrode. (A) Bare single carbon fiber electrode, (B) MXene-modified SCFE, and (C) MXene structure of the electrode.

This study is designed for minimal sample detection by single carbon fiber electrodes (SCFE) and pathogen DNA detection in breast milk. To increase the electrode surface, we modified the single carbon fiber electrode with MXene layers. MXenes are multifunctional residual nanomaterials that can increase the surface area. Then, the MXene-modified electrode surface was activated to form activated carbon groups for dCas9-sgRNA immobilization. dCas9 proteins are positively charged proteins; therefore, both protein immobilization via carbonyl groups and fluorine groups positively affect the immobilization that was carried out in a short time. The whole process was observed by EIS in Figure 1 and SEM pictures in Figure 2. Figure 1 shows the modification of the surface by MXene and CRISPR technology. SCFE shows higher conductivity than the modifications (black). Afterward, MXene modification provided more negative charges such as OH and F groups; therefore, the EIS curve (blue) increased. That increase is because the negatively charged redox probes were repelled by the negatively charged MXene layers. Positively charged protein and sgRNA immobilization decreased the EIS curve by shadowing negative charges, as shown in the red EIS curve. In this study, we applied a 5 mV AC potential to prevent an arc between layers of the MXene. The characteristics of the impedance curves show the Randles circuit model. In this model, the EIS spectrum’s beginning point was not at the origin of the EIS diagram, which means the redox probe resistance (Rs). The semicircle of the EIS spectrum shows the impedance(resistance) of the electrode surface. The electrochemical transformation of the redox probe releases electrons. Those electrons move toward the SCFE. The resistance provided by the surface is called electron transfer resistance, in other words, impedance (Ret). Then, the linear line represents the mass transfer resistance (redox probe movement to the surface) as Warburg impedance (W). This circuit model was used to calculate impedance as ohms to compare concentrations of the pathogen DNA.

Supplementary Figure 2 displays the XPS survey spectra of MXene. As can be seen, the elements F, O, Ti, and C are observed. This characterization result after MXene synthesis shows that the synthesis was carried out successfully.

SEM images and an EDX scan show the MXene modification of the SCFE. Figure 2A shows the single carbon fiber electrode surface. Figure 2B,C shows the MXene modifications and MXene structure, respectively. Figure 2C shows the layers of the MXene as nanomaterial, and the modification of the electrode shows the MXene modifications. Elemental analysis shows the chemical composition of the biosensor. Electrode surface modification steps were also observed with FT-IR and Raman analysis (Figure 1). The characterization of the electrode modification steps was examined with FT-IR and Raman spectra taken after each step.

In FT-IR analysis, to show partial consumption as a result of the covalent interaction with NHS-EDC during protein immobilization, the H peak at 3400 cm^–1^—which was originally intended to reflect hydroxyl groups on MXene—was changed to a transmittance of 60%. The peak at 1600 cm^–1^ is generally attributed to C=C stretching vibrations in aromatic rings or conjugated systems, indicating the presence of unsaturated carbon structures. Since the structures to which three FT-IR spectra belong are carbon based, this peak is present in all three spectra. The OH group is crucial for the formation of stable linkages. Amide I peak at 1650 cm^–1^ and Amide II peak at 1550 cm^–1^ are introduced by the presence of dCas9 proteins. Amide I represents C=O stretching typical of the protein backbone, while Amide II corresponds to N–H bending. These are standard peaks for protein characterization in FTIR. The intensity of the C–O–C peak, which appeared at a wavelength of 1100 cm^–1^ after Mxene modification, increased after sgRNA-dCas9 modification. Typical of organic compound ether connections, this peak verifies the existence of certain cyclic or acyclic ether structures that may be a component of the spacer molecules or surface chemistry employed in modifications. The phosphate group peak at 1080 cm^–1^ reflects the P–O stretching vibrations in the phosphodiester linkages of RNA. This is a critical feature when RNA or DNA is present on the surface, providing evidence of successful immobilization of the guide RNA. Nucleoside base peak at 1480 cm^–1^ represents general absorption by the nucleobases in the RNA. These peaks are somewhat broad and overlapping due to the complexity and variability in base structures.

In Raman analysis, the D-band (disorder band) appears around 1350 cm^–1^ as indicative of disordered or amorphous carbon, and the G-band (graphite band) appears around 1580 cm^–1^ as indicative of graphitic or ordered carbon. These bonds seen in the Raman analysis belong to the carbon fiber electrode. MXene typically shows peaks around 200 to 700 cm^–1^ due to metal–carbide bonds. After mixing Mxene with Nafion, modification of the carbon fiber electrode was made with this mixture. Around 600 and 1000 cm^–1^, peaks showed MXene contribution to the structure. Peak around 1000 cm^–1^ belongs to Nafion, related to −SO3 groups. Shifts and broadening in the D and G bands as a result of dCas9 and sgRNA modification show the effect of functional groups.

**Figure 3:**
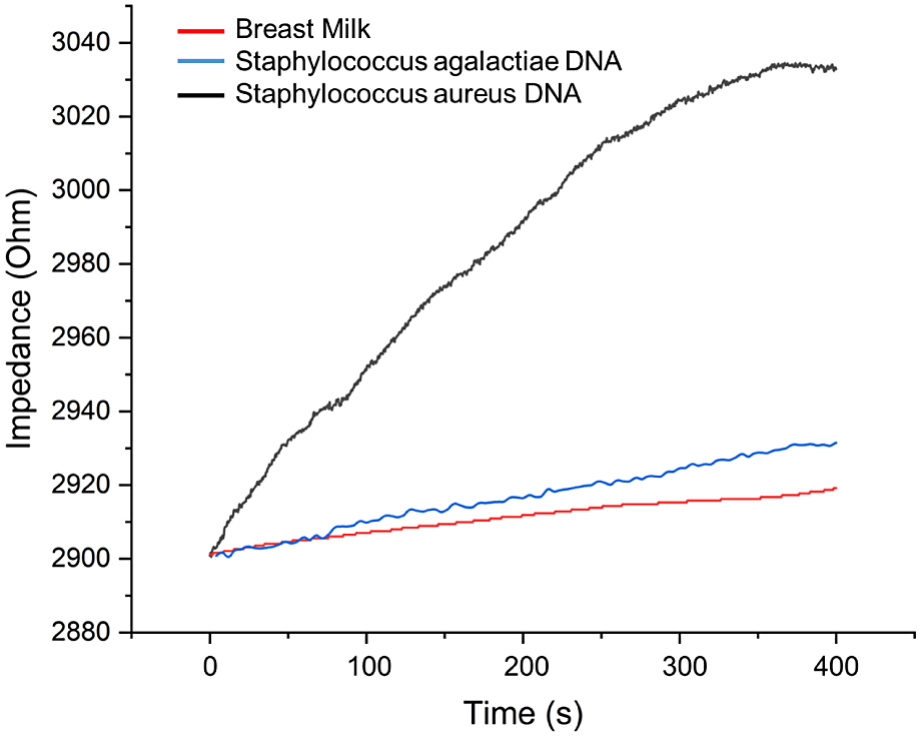
Chronoimpedimetric detection of biosensor for SAu (black line: SAu, blue line SAg, and red line breast milk as blank).

In Figure 1, red EIS was investigated by Bode plot to obtain the frequency of the chronoimpedance detection. The purpose of the detection is to optimize the detection time of the SAu DNA in breast milk, matrix effect, and selectivity. In breast milk, we used the most abundant pathogens and their DNAs. The method is useful for label-free binding of the biomolecules on the biosensor surface. The important point is to optimize frequency; the frequency was obtained from the Bode plot of the biosensor, where the phase angle is a plateau and impedance increases. That value is 600 Hz. 150 mV DC was chosen to prevent oxidation of the biomolecules in breast milk to avoid interference. As can be seen in Figure 3, chronoimpedimetric detection was used to obtain this information. The high selectivity of the CRISPR technology provided a fast response for the detection of SAu DNA in 30 s. The enzymatic properties of dCas9 decreased the response time. Figure 2 shows the SEM and EDX analysis of the single fiber electrode surface and MXene characterization. As can be seen in the figures, especially, single carbon fiber modified by MXene shows the Ti, C, and Al for the MXene surface, and we can consider P groups as the backbone of the sgRNA with the dCas9 protein. Therefore, our immobilization method was successfully performed. The modified electrode was then soaked in 500 μL of breast milk sample, SAu, and SAg DNAs separately. Chronoimpedance was performed by applying 150 mV AC, 5 mV AC, and 600 Hz for 400 s. AC potential was applied at 5 mV to avoid arcs between sharp edges of the MXene^25−28^ (Figure 2C). Those arcs under the alternative current can disrupt biological molecules. In Figure 3, blue and red lines show the selectivity against the SAu DNA’s detection (black). dCas9 is an enzyme that can break hydrogen bonds between DNA but has no endonuclease activity. Therefore, it can only bind the target DNA. Specificity to substrate shows high affinity increasing in seconds for SAu DNA. Chronoimpedance was performed for 400 s to investigate and optimize detection time. The detection time was obtained by 60 s in the real sample. Figure 3 also shows that the selectivity of the target DNA and other biomolecules or the other pathogen DNA does not affect the signal increase.

**Figure 4:**
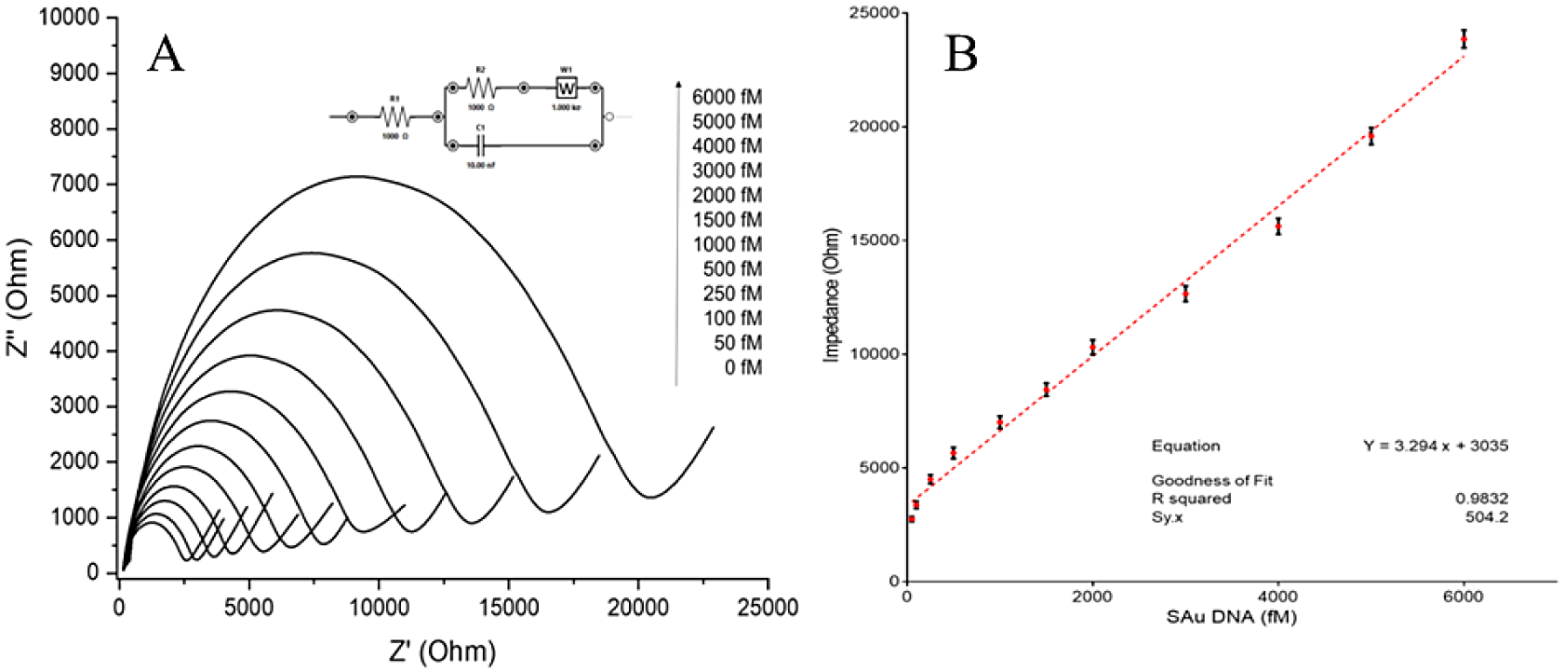
Calibration curve of the SAu biosensors. (A) Calibration curves by showing EIS values. (B) Calibration curve concentration (fM) versus impedance (*y* = 3.294 + 3035, *R*^2^ = 0.9832).

After the 60 s was obtained, a calibration curve was prepared. After every 60 s, biosensor surface was measured by EIS as shown in Figure 4A. Linearity was observed between 50 and 6000 fM; we prepared six different biosensors for the assessment of the biosensor reproducibility, and *R*^2^ was found as 0.9832 ± 0.0062 (CV%: 0.63%). Sy.x is also calculated as 504.2, which can be used to mathematically correlate the calibration curve by making the value 0.9832 (*R*^2^) zero. As a result, the goodness of the fit is also good. This shows quite a good reproducibility; however, ultralow concentrations provided higher standard deviations because of the sensitivity of single fiber electrode and nanomaterial of the structure of the biosensor. The data obtained in Figure 4A were calculated by fitting the EIS curves for the circuit model that is shown in Figure 4B. This circuit model is the Randles circuit model that includes R1 and R2 as redox solution and working electrode impedances, respectively. W represents the mass transfer resistance of Warburg impedance, and C shows the surface double-layer capacitance; however, because of the homogeneous structure, we used a constant phase element as Q. The *R*^2^ values give the EIS data by comparing the concentrations of the SAu DNA (Figure 4B). According to the six calibration curves, LOD and LOQ were calculated as 14.5 fM and 43.9 fM, respectively. Sensitivity is because of the single carbon fiber sensitivity and the increased surface area by MXenes on the electrode. LOQ is very close to the calibration curve beginning point, which shows the calibration curve is mathematically good. The lowest concentrations were reached by the dCas9 for the SAu DNAs.

**Figure 5:**
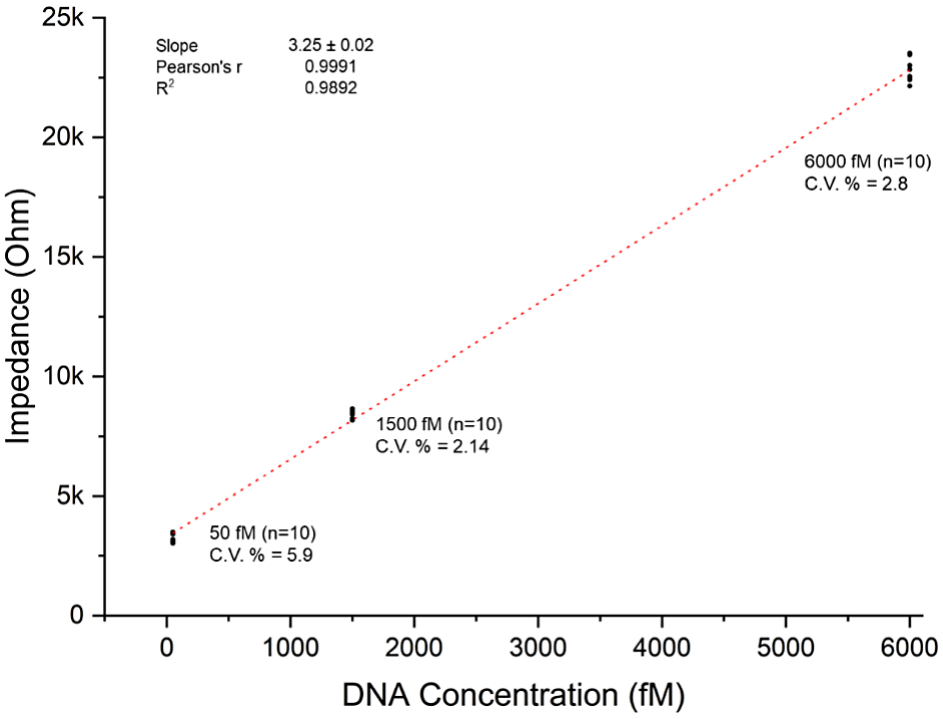
Repeatability and reproducibility tests of the biosensor (50, 1500, and 6000 fM).

**Figure 6:**
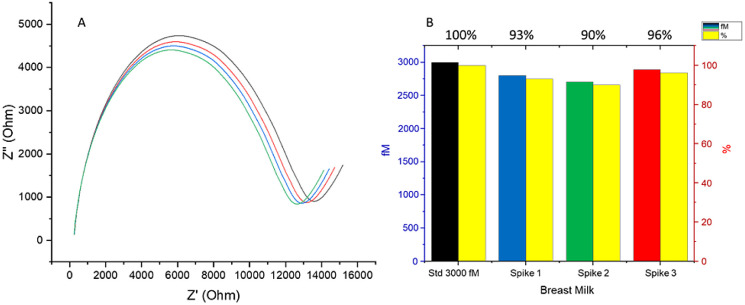
SAg biosensor for SAu DNA detection for 3000 fM (yellow colors show the percentage of the signal changes, and the other colors show the trials of fM concentration of DNA). (A) EIS spectra (ohm). (B) Relative signal changes (%).

**Figure 7:**
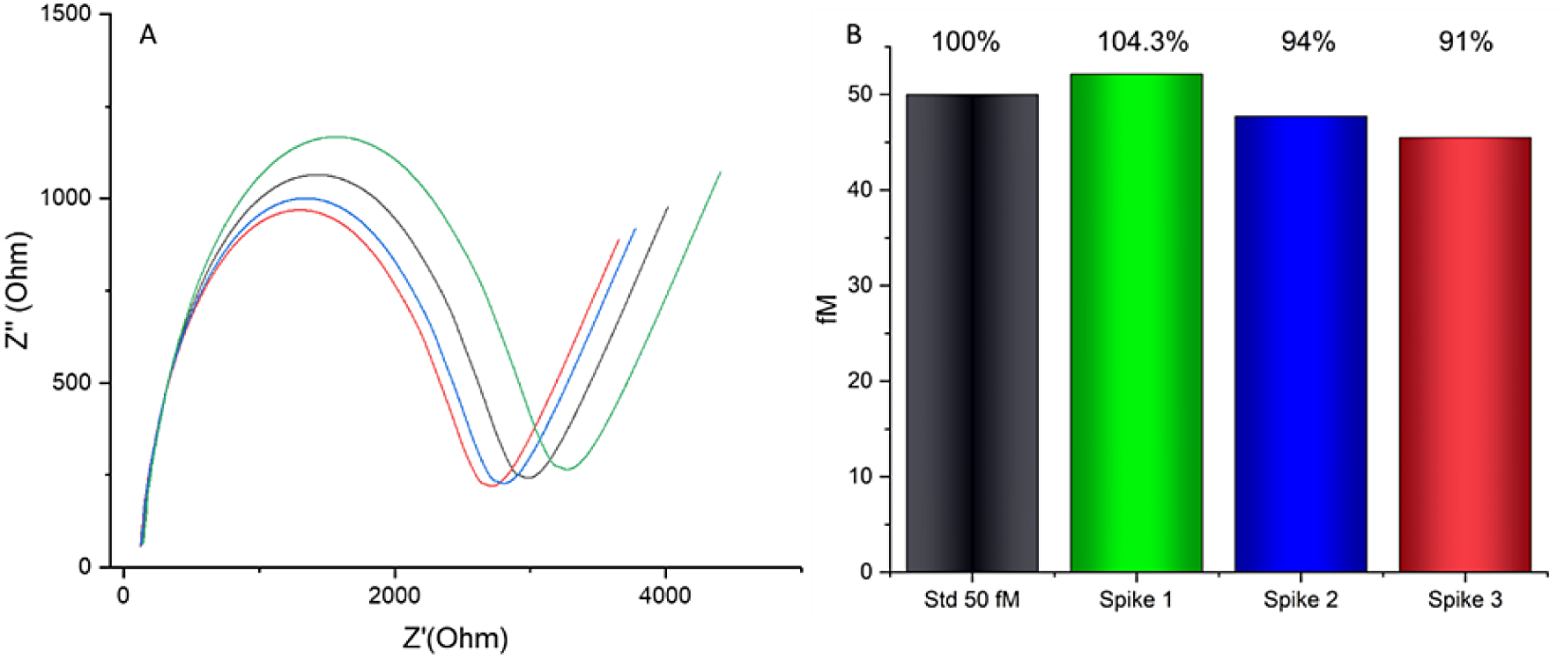
Recovery tests of the SAu biosensor for 50 fM DNA spiked in breast milk (black: calibration curve EIS 50 fM; the other colors are spikes as 50fM in breast milk: green is spike 1, blue is spike 2, and red is spike 3). (A) EIS spectra (ohm). (B) Relative signal changes (%).

**Figure 8:**
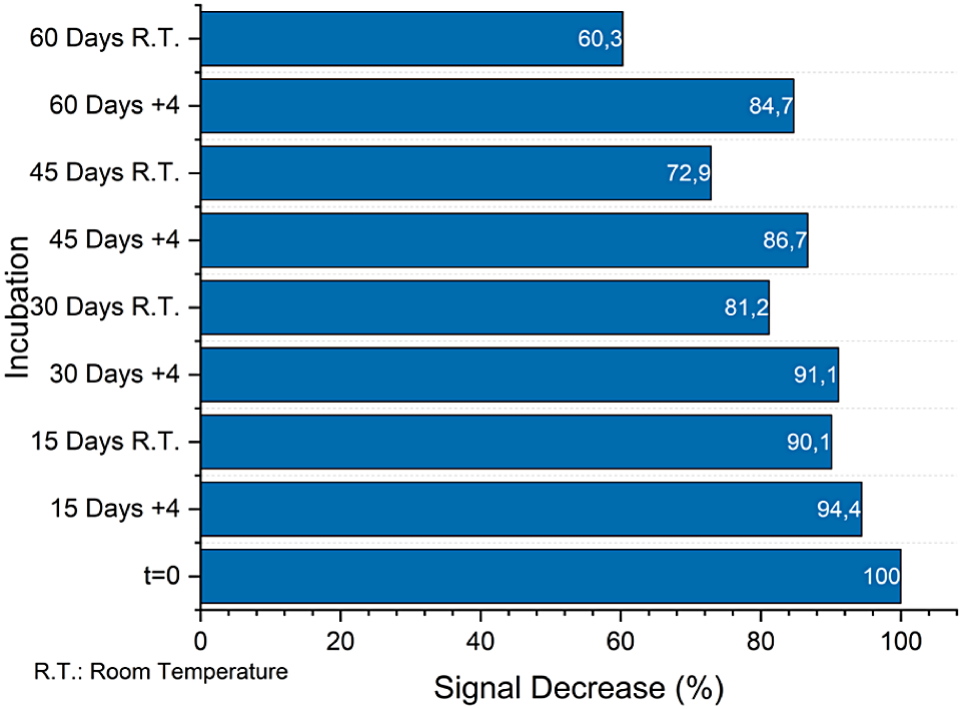
Storage stability tests of the biosensor (R.T.: room temperature, + 4:4 °C).

In this study, we evaluated electrode selectivity, repeatability, and real sample detection at high concentrations and the lowest concentration of the calibration curve. The evaluation encompassed a series of tests, including repeatability assessments at various concentrations, selectivity comparisons in the presence of complex matrices, and recovery tests, alongside an examination of storage stability under different conditions. Repeatability and reproducibility were rigorously tested using concentrations of 50 fM, 1500 fM, and 6000 fM. Impedance results of the repeatability and reproducibility for those concentrations as EIS spectra were given Supplementary Figures 3–5. The results, as depicted in Figure 5, demonstrated consistent detection across the tested range, underscoring the biosensor’s reliable performance. This consistency was further evidenced by the absence of significant variance in repeated trials, both under standard conditions and when samples were introduced to a breast milk matrix at the 3000 fM concentration (Figure 6). These findings affirm the biosensor’s robustness and precision in repeated measurements. Selectivity was scrutinized by preparing the SAg biosensor using the same methodology applied to the SAu biosensor, enabling a direct comparison. Notably, the application of a 3000 fM standard without the breast milk matrix, alongside a sample with 3000 fM integrated into breast milk, yielded no observable increase in signal for either the standard or the sample trials. This outcome illustrates the biosensor’s high selectivity and sensitivity, even in the presence of complex biological matrices. The reproducibility of these results across different electrodes further supports the biosensor’s reliability, attributed to a stable surface modification technique that ensures consistent immobilization and equal binding sites at every step. The recovery test involved introducing a 50 fM concentration, corresponding to the biosensor’s lowest detection limit, into breast milk. This was directly compared to the standard measurement. The observed matrix effect had an average impact of 4%, as presented in Figure 7. This effect correlates closely with the repeatability assessment at 50 fM in Figure 5, which indicated a repeatability rate of approximately 5%. These results collectively validate the minimal influence of the matrix on the biosensor’s performance. The final optimization phase focused on storage stability, with findings revealing the biosensor’s commendable durability over time (Figure 8). Notably, cold storage conditions were found to preserve the stability of the protein and its MXene coating more effectively than storage at room temperature. This distinction underscores the importance of temperature control in extending the biosensor’s storage life and maintaining its performance integrity.

The LOD values of this study and some studies in the literature are given in Table 1.

**Table 1:** DNA-Based Biosensors for *Staphylococcus aureus*^a^

method	electrode	detection limit	reference
DPV*	MWCNT–Chi and bismuth/tDNA	2.44 × 10 ^–14^ M	(29)
diffusometry	AuNPs/MRSA/ssDNA	1x 10^–11^	(30)
DPV	dsDNA/CTS–Co_3_O_4_–GR/CILE	4.3 × 10^–13^ M	(31)
EIS	AuE/H-(CH_2_)_6_-ssDNA/Cas12a/gRNA	3x 10^–9^ M	(32)
EIS	SCFE-MXene-dCas9-sgRNA	1,45x 10^–14^ M	this work

a Differential pulse voltammetry.

## Conclusions

This study presents a significant advancement in the field of biosensing, particularly for the detection of pathogen DNA in complex biological samples, such as breast milk. By employing single carbon fiber electrodes (SCFE) modified with MXene layers, this research demonstrates a novel approach to enhance the electrode surface area and thereby increase biosensor sensitivity and specificity. The activation of the MXene-modified electrode surface to form carbonyl groups, followed by the immobilization of dCas9-sgRNA, capitalizes on the unique properties of these materials and biological molecules for efficient pathogen detection. The utilization of electrochemical impedance spectroscopy (EIS) and scanning electron microscopy (SEM) provided detailed insights into the electrode modifications and the immobilization process, affirming the successful enhancement of the biosensor’s performance. The chronoimpedimetric detection method, optimized for the specific detection of SAu DNA in breast milk, illustrates the potential of this technology to achieve rapid, selective, and sensitive pathogen detection, with a notable detection time of 60 s in real samples. Future perspectives of this research could focus on several areas to further advance this promising technology. First, expanding the range of detectable pathogens beyond SAu DNA could significantly broaden the applicability of this biosensor in clinical diagnostics and public health monitoring. Investigating the biosensor’s performance with other biological fluids or environmental samples could also extend its utility across different domains. Second, further miniaturization and integration of the biosensor into portable or wearable devices could facilitate real-time, on-site pathogen detection, greatly benefiting remote or resource-limited settings. This could also open avenues for continuous health-monitoring applications. Third, exploring other nanomaterials or surface modifications could unveil new pathways to enhance biosensor performance even further, potentially lowering detection limits and reducing response times. Integrating machine learning algorithms for data analysis might also improve the specificity and sensitivity of pathogen detection, particularly in complex sample matrices. Lastly, conducting long-term stability and reproducibility studies under various storage conditions and in real-world applications would be crucial for the transition of this technology from the laboratory to the market. Addressing scalability and cost-effectiveness will also be key factors in determining the success of this biosensor technology in clinical settings and beyond. In summary, this study lays a strong foundation for the development of advanced biosensing technologies for pathogen detection. With further research and development, the approaches and insights gained here could lead to widespread applications in healthcare, environmental monitoring, and beyond, significantly impacting public health and safety.

## Data Availability

Data will be made available upon request.

## Supporting Information Available

FTIR and Raman analysis of dCas9-sgRNA-MXene-SCFE biosensor; XPS survey spectra of the Mxene structure; EIS spectrums of 50fM target DNA analysis for repeatibility and reproducibility tests; EIS spectrums of 1500fM target DNA analysis for repeatibility and reproducibility tests; EIS spectrums of 6000fM target DNA analysis for repeatibility and reproducibility tests(PDF)
