# Supplementary material for: Impedimetric Single Carbon Fiber Electrode for Ultrasensitive Detection of Staphylococcus aureus Pathogen DNAs in Breast Milk by CRISPR Technology
Source: ACS Omega. 2024 May 24;9(23):25172–80. doi: 10.1021/acsomega.4c02738 (PMC11170623; doi:10.1021/acsomega.4c02738)
Supplement: Supplementary file 1 — ao4c02738_si_001.pdf [file ao4c02738_si_001.pdf]

Impedimetric Single Carbon Fiber Electrode For Ultrasensitive Detection Of Staphylococcus Aureus Pathogen DNAs In Breast Milk By CRISPR Technology

Hilmiye Deniz ERTUĞRUL UYGUN[1]*, Dilek ODACI[2]

[1]Dokuz Eylül University, Center for Fabrication and Application of Electronic Materials, Buca, İzmir, Türkiye

[2]Ege University, Faculty of Science, Department of Biochemistry, Bornova, İzmir, Türkiye

Supplementary file

Supplementary Figure 1. FTIR and RAMAN analysis of dCas9-sgRNA-MXene-SCFE biosensor

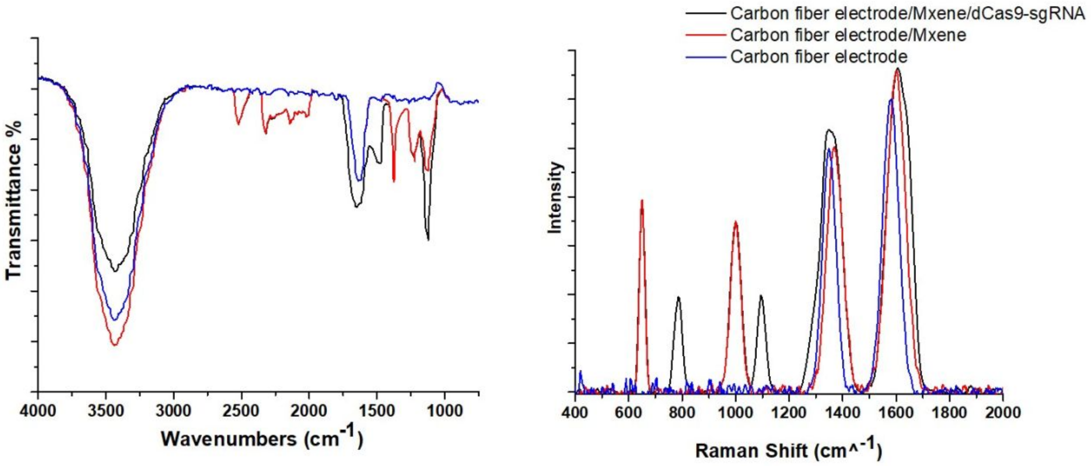

Supplementary figure 2. XPS survey spectra of the Mxene structure.

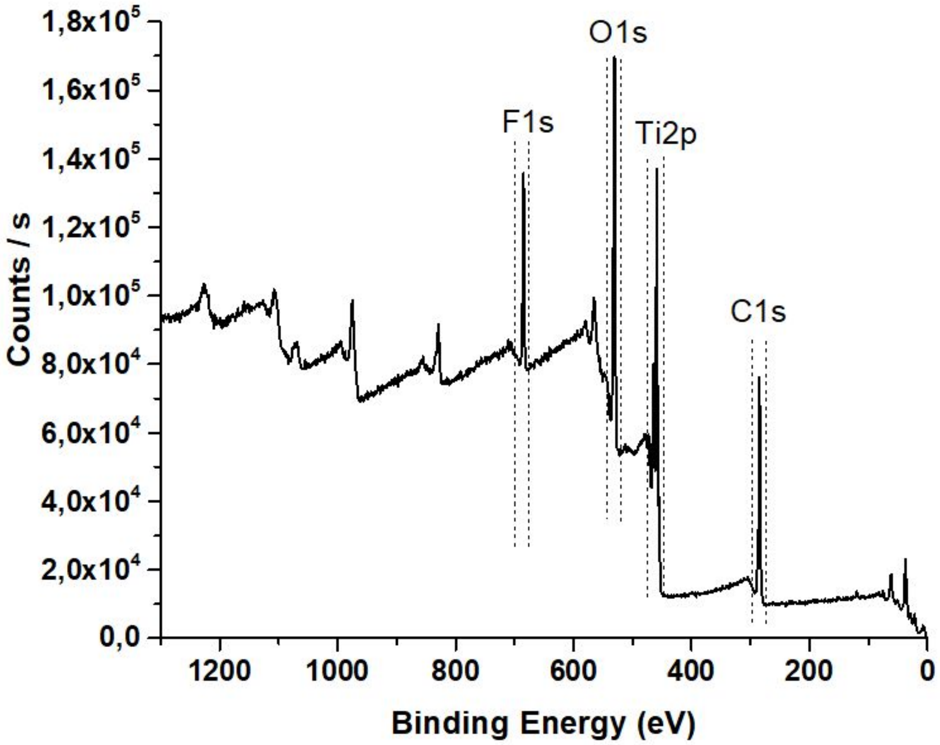

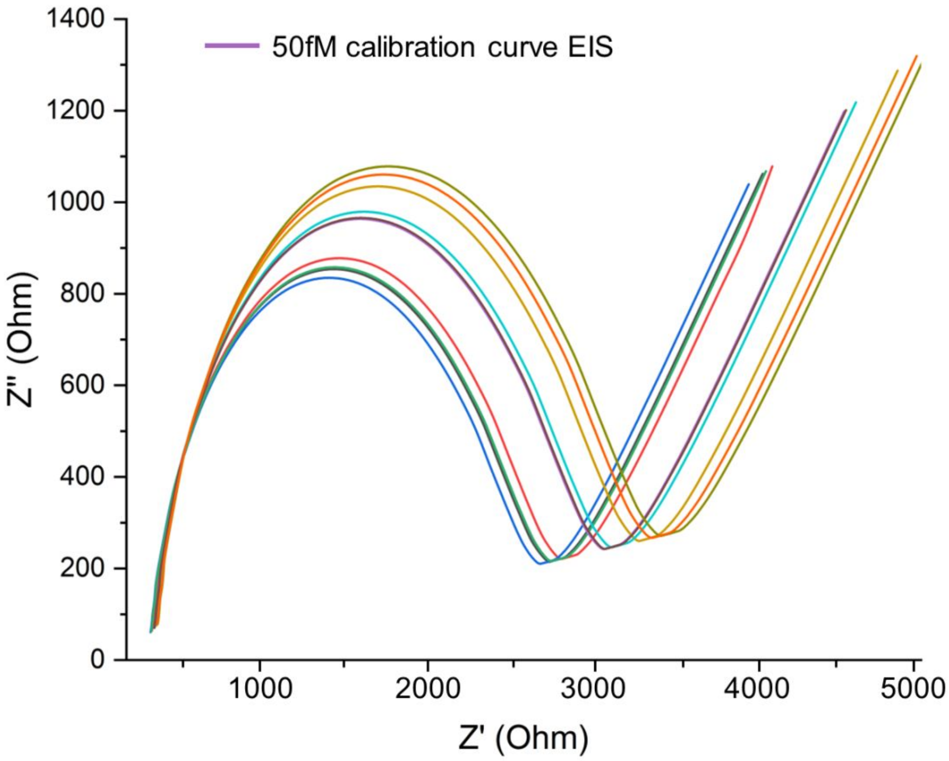

Supplementary figure 3. EIS spectrums of 50fM target DNA analysis for repeatibility and reproducibility tests (n=10)

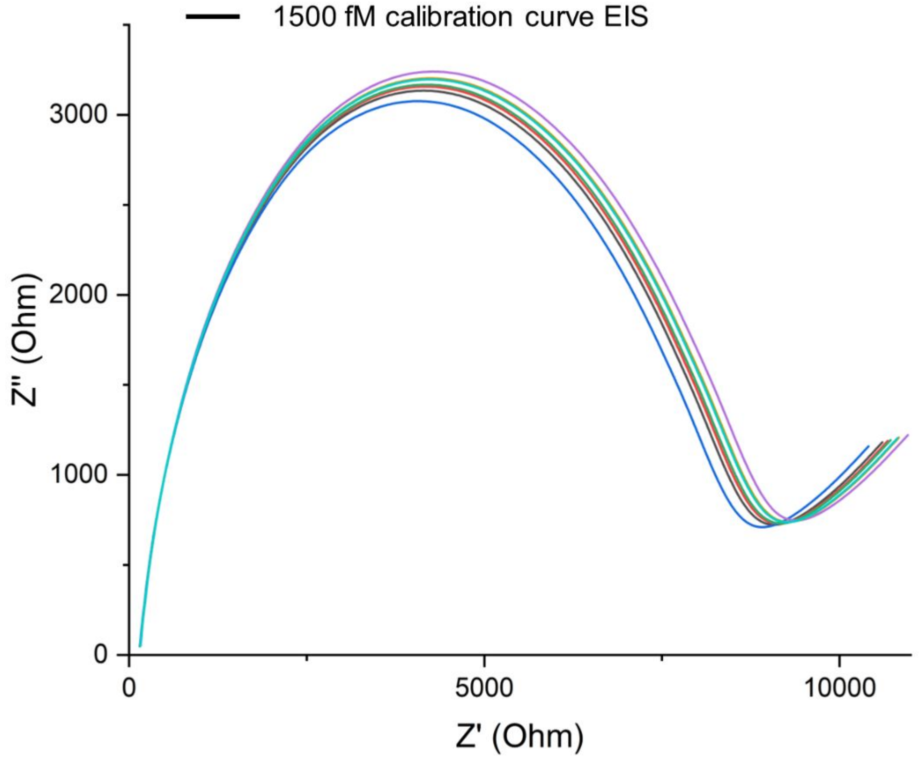

Supplementary figure 4. EIS spectrums of 1500fM target DNA analysis for repeatibility and reproducibility tests (n=10)

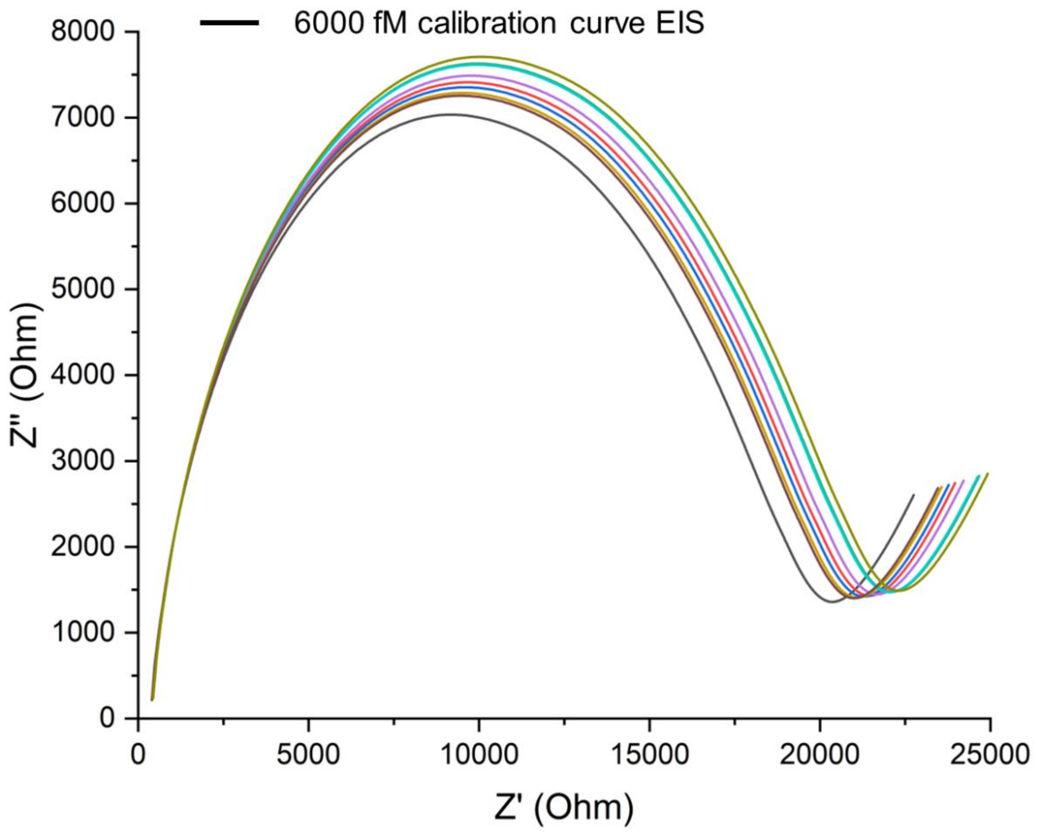

Supplementary figure 5. EIS spectrums of 6000fM target DNA analysis for repeatibility and reproducibility tests (n=10)
